# The Role of the Eph Receptor Family in Tumorigenesis

**DOI:** 10.3390/cancers13020206

**Published:** 2021-01-08

**Authors:** Meg Anderton, Emma van der Meulen, Melissa J. Blumenthal, Georgia Schäfer

**Affiliations:** 1 International Centre for Genetic Engineering and Biotechnology (ICGEB) Cape Town, Observatory, Cape Town 7925, South Africa; andmeg005@myuct.ac.za (M.A.); vmlemm001@myuct.ac.za (E.v.d.M.); 2 Institute of Infectious Disease and Molecular Medicine (IDM), Faculty of Health Sciences, University of Cape Town, Observatory, Cape Town 7925, South Africa; 3 Division of Medical Biochemistry and Structural Biology, Department of Integrative Biomedical Sciences, Faculty of Health Sciences, University of Cape Town, Observatory, Cape Town 7925, South Africa

**Keywords:** Eph receptors, EphA2, Kaposi’s sarcoma, endothelial cells

## Abstract

**Simple Summary:**

The Eph receptor family is implicated in both tumour promotion and suppression, depending on the tissue-specific context of available receptor interactions with ligands, adaptor proteins and triggered downstream signalling pathways. This complex interplay has not only consequences for tumorigenesis but also offers a basis from which new cancer-targeting strategies can be developed. This review comprehensively summarises the current knowledge of Eph receptor implications in oncogenesis in a tissue- and receptor-specific manner, with the aim to develop a better understanding of Eph signalling pathways for potential targeting in novel cancer therapies.

**Abstract:**

The Eph receptor tyrosine kinase family, activated by binding to their cognate ephrin ligands, are important components of signalling pathways involved in animal development. More recently, they have received significant interest due to their involvement in oncogenesis. In most cases, their expression is altered, affecting the likes of cell proliferation and migration. Depending on the context, Eph receptors have the potential to act as both tumour promoters and suppressors in a number of cancers, such as breast cancer, colorectal cancer, lung cancer, prostate cancer, brain cancer and Kaposi’s sarcoma (KS), the latter being intrinsically linked to EphA2 as this is the receptor used for endothelial cell entry by the Kaposi’s sarcoma-associated herpesvirus (KSHV). In addition, EphA2 deregulation is associated with KS, indicating that it has a dual role in this case. Associations between EphA2 sequence variation and KSHV infection/KS progression have been detected, but further work is required to formally establish the links between EphA2 signalling and KS oncogenesis. This review consolidates the available literature of the role of the Eph receptor family, and particularly EphA2, in tumorigenesis, with the aim to develop a better understanding of Eph signalling pathways for potential targeting in novel cancer therapies.

## 1. Introduction

The Eph family of receptor tyrosine kinases (RTKs), involved in signalling pathways that are key to embryogenesis and tissue patterning, have been implicated in the oncogenesis of a number of cancers. Generally, this involves their aberrant expression, allowing them to act as either tumour promoters or tumour suppressors, depending on the context [1,2]. Here, the focus is on the role of Eph receptors in breast cancer, colorectal cancer, lung cancer, prostate cancer and brain cancer as these have been the most extensively researched and are among the most common and/or debilitating cancers known. In addition, more recent evidence for the involvement of Eph receptors in Kaposi’s sarcoma (KS), the most common acquired immune deficiency syndrome (AIDS)-related malignancy worldwide, has been investigated [3]. Understanding the oncogenic mechanisms of Eph receptors, however, proves to be a challenge due to the fact that both canonical and noncanonical pathways exist. The most well-characterised example of this is for EphA2, where the classical ligand- and tyrosine kinase-dependent signalling mechanism is accompanied by a pathway in which tumour promotion is achieved independently of ligand or tyrosine kinase activation of the receptor [4]. Resolving these distinct pathways is, therefore, necessary if the results of functional studies are to be understood.

## 2. Eph Receptor Structure and Signalling

**Figure 1:**
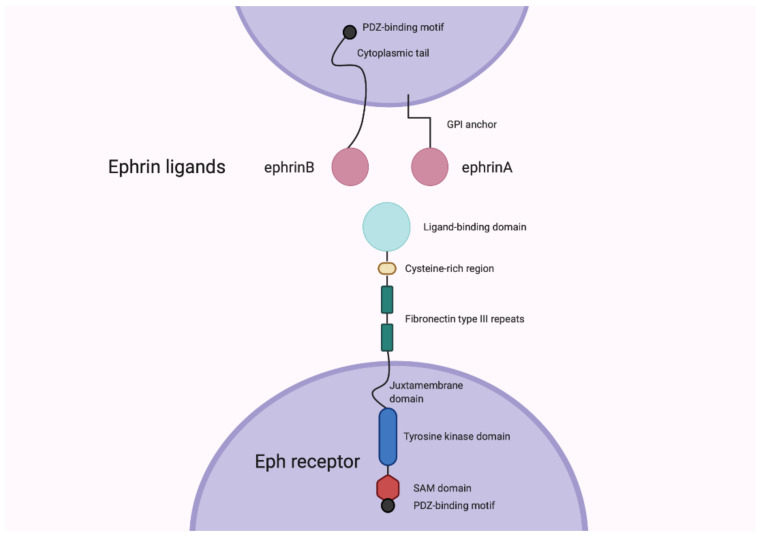
The general structure of the Eph receptors and ephrin ligands. Both the ephrin-A and ephrin-B ligands are depicted here. Figure created with BioRender.com.

Eph receptors are type-I transmembrane proteins with a structure that is generally conserved. The ligand-binding domain, cysteine-rich region and two fibronectin type III repeats compose the extracellular domain of the receptor, while the intracellular region is made up of a juxtamembrane domain, a protein tyrosine kinase (Pkinase-Tyr) domain, a sterile alpha motif (SAM) and a C-terminal PDZ-binding motif (Figure 1) [2,5]. There are two classes of Eph receptors, grouped according to the ligands they preferentially bind. While there are a few exceptions, EphA-type receptors bind ephrin-A ligands and the EphB-type receptors bind ephrin-B ligands (Figure 1) [5]. The ephrin ligands are generally membrane-bound, and it is the difference in anchorage that distinguishes the two classes. Ephrin-A ligands are attached to the membrane via a glycophosphatidylinositol anchor; this is in contrast to the ephrin-B ligands, which have a transmembrane domain, as well as a cytoplasmic region with a PDZ domain [5,6]. Heterodimerisation upon interaction between an Eph receptor and its ephrin ligand is followed by the formation of tetrameric complexes, leading to receptor tyrosine phosphorylation and kinase activation [6]. A unique feature of Eph-ephrin signalling is that it is bidirectional. Conventional forward signalling is that already mentioned, in which the signal is transduced in the receptor-expressing cell. Reverse signalling, on the other hand, involves a signal transduction cascade in the ephrin-expressing cell. For example, upon Eph receptor engagement, the cytoplasmic tail of the ephrin-B ligand becomes tyrosine phosphorylated and can then interact with signalling molecules that contain SRC-homology-2 domains [5,6,7].

This signalling plays a role in a number of biological processes important for both development and homeostasis. By modifying cell adhesion and the organisation of the actin cytoskeleton, Eph signalling controls cell morphology and migration. Eph signalling also affects cell proliferation and differentiation [7,8]. Many of these functions are also important in cancer development, when well-controlled functions become dysregulated. Hence, in addition to their expression in normal tissues, Eph receptors are expressed in cancer cells and the tumour microenvironment where they are involved in processes related to tumorigenesis and metastasis [6,8,9]. Expression in tumours, however, is not always increased, and the downregulation of certain Eph molecules in a number of malignancies suggests that Eph receptors can act as both tumour promoters and suppressors [2,8]. In the following sections, the role of Eph receptors in breast cancer, colorectal cancer, lung cancer, prostate cancer, brain cancer and KS is discussed and summarised in Table 1, providing a synthesis of the literature that has been published to date.

## 3. EphA2 and EphB4 Are the Main Oncogenic Eph Family Members in Breast Cancer

The best characterised Eph receptors in breast cancer are EphA2 and EphB4, but there are also others that have been found to play a role. EphA2, the main EphA receptor to have been extensively studied for its involvement in breast carcinomas, is overexpressed in 40% of breast cancers and is generally correlated with a poor prognosis [10,49]. This overexpression has been shown to be linked to transformation of mammary epithelial cells, mediating cancer cell migration in culture and inducing tumour formation upon injection of these cells into nude mice [6,9]. Conversely, a knockdown of EphA2 resulted in a reduction in tumorigenicity in human breast cancer cells [9]. The ability of the overexpressed EphA2 to cause oncogenic transformation appears to be dependent on a low level of ligand-induced forward signalling, as the receptor is poorly tyrosine phosphorylated (see Section 9) in breast cancer cell lines, and ligand binding reversed the malignant phenotype of the cells conferred by EphA2 overexpression [9,50]. It has also been proposed that low EphA2 forward signalling is a result of dephosphorylation of the receptor by the low-molecular-weight phosphotyrosine phosphatase (LMW-PTP) as this has been shown to cause transformation of mammary epithelial cells [51]. Alternatively, loss of E-cadherin has been suggested as this leads to an impairment of the EphA2-ephrin-A1 interaction, a phenomenon that was detected in malignant breast cancer cells [52]. Another scenario proposed by Brantley-Sieders et al. involves the possibility that EphA2 exerts noncanonical tumour-promoting effects via crosstalk with oncogenic signalling pathways, entirely independent of ephrin ligand stimulation [11]. An example of this is its complex formation with ErbB2, leading to enhanced Ras-MAPK and Rho GTPase signalling. These pathways are involved in cell proliferation and cell motility, respectively, and so this interaction may contribute to tumour progression [49].

The expression of EphA4 and EphA7 receptors was also found to be upregulated in breast cancer, which correlated with a worse prognosis [11]. EphA4, in particular, has been shown to be associated with proliferation, migration and invasion of breast cancer cells, in the context of transforming growth factor-beta (TGFb) signalling [53,54]. Similarly, the expression of EphA10 was associated with lymph node metastasis in breast cancer [13]. EphA10, however, is a kinase-deficient receptor, and so it has been hypothesised that it exerts its effects through an interaction and nuclear colocalisation with EphA7, the consequences of which could be the transcriptional activation of genes involved in invasiveness [55]. EphA5, on the other hand, was found to be downregulated in breast cancer cell lines, associated with hypermethylation of its promoter. This suggests that it may play a role as a tumour suppressor and could be of use as a prognostic marker [12].

Similar to EphA2, EphB4 has been implicated in breast cancer in numerous studies. Its expression has been found to be both upregulated and downregulated in breast cancer cells, suggesting it has the ability to be both pro- and antioncogenic [2]. When stimulated by its preferred ligand, ephrin-B2, EphB4 behaved as a tumour suppressor in a mouse xenograft model of breast cancer [56]. This activation of EphB4 triggered the Abl-Crk pathway, blocking cell proliferation and leading to downregulation of matrix metalloproteinase-2 (MMP2), which is proinvasive [56,57]. Accordingly, high levels of EphB4 expression in breast cancer cell lines was found to be often accompanied by low expression of its ligand, ephrin-B2, and this allows for signalling via ligand-independent pathways and evasion of EphB4’s tumour-suppressing effects [56,58]. Complicating matters further, it has been reported that EphB4 can exert tumour-suppressor effects independent of ligand stimulation, e.g., through a decrease in integrin-mediated adhesion [59]. In MCF-7 breast cancer cells, on the other hand, EphB4 displayed pro-oncogenic effects, via ephrin-B2-mediated activation of the extracellular signal-regulated kinase (ERK) pathway, which has been linked to the promotion of protein phosphatase [60]. Higher levels of EphB2 expression were associated with poor survival in breast cancer patients, suggesting it has prognostic value [14]. It was shown that EphB2 was regulated by TGFb signalling, which could be inhibited by p53 [61]. However, EphB2’s role in the different stages of breast cancer is not explicit and a model to resolve this has been suggested, i.e., in non-invasive cells, EphB2 stimulates autophagy, which triggers apoptosis, but when apoptosis is blocked in cancer cells, autophagy has a prosurvival role instead, leading to the promotion of invasion [62].

EphB6, a receptor lacking kinase activity, thereby considered “kinase dead,” has been shown to be downregulated in breast carcinoma cells, suggesting a role as a tumour suppressor [15]. In invasive breast carcinoma cells, EphB6 expression was found to be transcriptionally silenced by promoter hypermethylation, and it has, therefore, been proposed as a biomarker for breast cancer detection and diagnosis [15].

## 4. Eph Receptors Are Downregulated in the Advanced Stages of Colorectal Cancer

In slight contrast to the distinct upregulation or downregulation already mentioned in breast cancer (see Section 3), the expression levels of EphA1 and EphA2 were found to differ between the stages of colorectal cancer. In stage I and the locally invasive stage II, overexpression of EphA1 and EphA2 has been observed. This was followed by a downregulation in the metastatic stage III via epigenetic silencing in which there was increased methylation of CpG islands in the gene’s promoter [16]. In support of this, lower expression of EphA1 was correlated with shorter survival in colorectal cancer patients [16]. In order to establish the link between this decreased EphA1 expression and increased invasiveness, EphA1 was knocked out in HRT18 colorectal cancer cells using the CRISPR-Cas9 genome editing system, resulting in increased spreading and adhesion of the cancer cells, which suggests that EphA1 may be able to suppress these processes, achieved through the deactivation of the ERK and JNK signalling pathways [63].

It has been shown that EphA7 expression is lost in colorectal cancer, also as a result of epigenetic silencing mediated by CpG methylation of the promoter [19]. Similarly, EphA3 expression was downregulated in colorectal cancer cells, possibly via the same mechanism, and suggestive of the fact that EphA3 may possess tumour-suppressing abilities [17].

Finally, in the progeny of colorectal cancer cells subjected to radiation treatment, EphA4 activation was shown to be induced. This EphA4 overexpression caused a reduction in E-cadherin expression and, therefore, disrupted cell–cell adhesion; it also activated the ERK1/2 pathway, which led to increased cell migration and invasion [18].

As in the case of EphA1 and EphA2, initial upregulation of EphB2 and EphB3 expression appears to be followed by a loss of expression in the more advanced metastatic stages of colorectal cancer. This upregulation was found to be a result of activating Wnt pathway mutations leading to constitutive transcription from the Tcf-4 complex; EphB2 and EphB3 are Tcf-4 target genes, and their expression is thereby enhanced [20,21]. The tumour-suppressor function of EphB2 and EphB3 is thought to be reliant on their ability to compartmentalise tumour cells, which involves E-cadherin-mediated adhesion [21]. The adenoma-carcinoma transition is generally associated with a loss of EphB2 and EphB3 expression and this explains how the cells can then invade the surrounding tissue [1,20].

Conversely, the expression of EphB4 is upregulated in colorectal cancer, suggesting that it functions as a tumour promoter [19]. By overexpressing EphB4 in colorectal cancer cell lines using EphB4 expression vectors, vascularisation and migratory ability were enhanced [64]. Conversely, a loss of EphB6 expression in colorectal cancer has been found to be correlated with poor prognosis contributing to increased metastasis [22,23].

## 5. The Overexpression of Many Eph Family Members Promotes Lung Cancer Tumorigenesis

The expression levels of EphA1, EphA4, EphA5 and EphA7 have been investigated in patients with nonsmall cell lung carcinoma (NSCLC), the most common type of lung cancer. Giaginis et al. suggested that the increased expression detected in non-advanced stage NSCLC was an indication that these receptors may participate in the biological mechanisms underlying carcinogenic evolution [24]. Notably, higher levels of EphA4, EphA5 and EphA7 have been associated with favourable patient survival, and the authors suggest they may, therefore, be of use as biomarkers for prognosis [24]. For EphA2, higher levels were found to be correlated with more advanced stages of NSCLC compared to earlier stages, and increased EphA2 expression was associated with the development of brain metastasis [25]. A G391R mutation in the EphA2 gene was recurrent in lung squamous cell carcinoma and associated with increased phosphorylation of two serine residues within mTOR—it has been suggested that this may be functionally important for EphA2’s invasive signals [65]. Low expression of EphA3 in small cell lung cancer (SCLC) has been associated with multidrug resistance [26]. Inducing EphA3 overexpression reduced the phosphorylation of components of the phosphoinositide 3-kinase (PI3K)/BMX/signal transducer and activator of transcription 3 (STAT3) signalling pathway, increased apoptosis and decreased chemoresistance, suggesting that EphA3 has a tumour suppressor role and could be a novel therapeutic target for SCLC [26].

Like EphA2, EphB3 was shown to be overexpressed in NSCLC in which it likely promotes cell growth and migration. Consequently, the loss of EphB3 lead to activation of caspase-8-mediated apoptosis and suppression of cell migration [27]. Stimulation of EphB3 by its ligand, ephrin-B1, resulted in suppression of NSCLC metastasis. The proposed mechanism includes EphB3 activation, followed by RACK1-mediated formation of a ternary complex of protein phosphatase 2a, Akt, EphB3 and RACK1, leading to inhibition of Akt phosphorylation and consequently inhibition of cell migration [27]. Because ephrin-B1 is not overexpressed in lung cancer, this tumour-suppressive signalling pathway is not common, but it has still been hypothesised as an opportunity for a novel therapeutic strategy [35,66]. EphB4 was also found to be overexpressed in lung tumours, promoting cellular proliferation, colony formation and motility. However, paradoxical to this, there seems to be a positive correlation between EphB4 expression and patient survival, and therefore, it was suggested to be of use as a positive prognostic indicator in lung cancer [28]. In contrast, EphB6 was shown to be tumour suppressive, which correlated with its lower expression in metastatic compared to nonmetastatic NSCLC. This downregulation was found to be due to epigenetic silencing mediated by hypermethylation of its promoter DNA [29,30]. Low expression of EphB6 was, therefore, suggested as a poor prognostic indicator for NSCLC [67]. In addition, mutations in EphB6 could potentially cause a loss of function in NSCLC. For example, an in-frame deletion mutation identified in a subset of NSCLC patients increased the metastatic capacity of NSCLC cells in an in vivo mouse model [29].

## 6. Eph Receptors Have a Potential Relevance in Prostate Cancer

While the expression levels of other EphA receptors seem to be upregulated in prostate cancer, the expression of EphA1 was shown to be downregulated, decreasing from normal prostate to primary prostate tumour cells and finally to metastatic cells, possibly due to CpG methylation of the promoter [6,31]. This suggests that it may have a role to play in preventing the transformation of cells [31]. In contrast, EphA2 expression increased as prostatic epithelial cells progressed toward a more aggressive phenotype. Importantly, EphA2 expression was increased in high-grade prostatic intraepithelial neoplasia, the precursor to prostatic adenocarcinoma, indicating a possible role for EphA2 in the early stages of prostatic carcinogenesis [32]. Similarly, EphA3 was overexpressed in the more invasive cell lines, implicating it in the development of prostate cancer [31]. Comparable trends are apparent for EphA4; its importance has been highlighted through siRNA knockdown, which resulted in a reduction in prostate cancer cell viability [33]. Soler et al. suggest that the levels of EphA4 may be controlled by ERBB3/HER3, a prostate cancer-associated receptor, as knockdown of ERBB3 in DU-145 cells resulted in EphA4 downregulation [68]. In contrast, EphA7 has been assigned a tumour-suppressive role and was found to be downregulated in prostate cancer, the mechanism responsible for this being CpG methylation [37]. These tumour-suppressive abilities involve the enhancement of tumour cell apoptosis, which is dependent on ligand stimulation as EphA7 mutants that cannot be phosphorylated were unable to exert these same effects [69]. Similarly, EphA5 has been suggested to have a suppressive role in the progression of prostate cancer, highlighted by its downregulation in prostate cancer tissues and the fact that this was associated with higher Gleason scores [34]. Finally, EphA6 has been identified as a metastasis gene and positively correlated with the progression of prostate cancer, by facilitating invasiveness and angiogenesis [36]. EphA6 knockdown decreased Akt and thereby the PI3K/Akt pathway, which contributes to prostate cancer progression, as well as EIF5A2, a target gene for Akt, which promotes melanoma cell invasion; this indicates that EphA6 may mediate its effects via interaction with the PI3K/Akt pathway [35,36].

Support for EphB2 functioning as a tumour suppressor in prostate cancer comes from the identification of its mutational inactivation; when DU-145 cells were transfected with a wild-type EphB2, clonogenic growth was suppressed [38]. Conversely, EphB3 and EphB4 were upregulated in prostate cancer cells, possibly contributing to invasion and metastasis through the deregulation of contact inhibition of locomotion (CIL) as CIL was restored upon knockdown of the two receptors [39]. It has also been shown that EphB4 regulates the expression of the integrin β8 receptor and that this might be promoting prostate cancer cell motility [70].

## 7. Eph Receptors Are Potential Prognostic Markers in Glioblastoma and Medulloblastoma

Glioblastoma and medulloblastoma are two of the most aggressive cancers affecting the brain. In glioblastoma, overexpression of EphA2 was shown to correlate with poor prognosis [40]. Its tumour-promoting activities were found to be exerted via a ligand-independent mechanism involving the phosphorylation of EphA2’s serine 897 residue [40]. It has been shown that this phosphorylation event can occur either through the mitogen-activated protein kinase kinase 1 (MEK)/ERK/ribosomal S6 kinase (RSK) pathway or through the PI3K/Akt pathway upon the binding of epidermal growth factor (EGF) to EphA2-expressing cells [40,71]. EphA2 is part of a reciprocal regulatory loop with Akt and acts as an Akt substrate when phosphorylated at S897—with consequences for enhanced cell migration and invasion [71]. In addition, EphA2 can promote self-renewal and tumorigenicity of tumour-propagating cells in glioblastoma as these were suppressed upon siRNA-mediated knockdown of EphA2 expression [72]. In medulloblastoma, EphA2 was implicated in vasculogenic mimicry (VM) through its activation of PI3K, which regulates MMP-14 and subsequently activates MMP-2, thereby driving VM [73]. Overexpression of EphA3 has also been reported in glioblastoma and this resulted in inhibition of the MAPK pathway, keeping tumour cells in a dedifferentiated and tumorigenic state. This highlights its potential use as a therapeutic target in glioblastoma patients [41]. In addition, highly expressed in malignant gliomas, EphA4 forms a complex with fibroblast growth factor receptor 1 (FGFR1), accelerating the canonical FGFR1 signalling pathway and resulting in increased cell proliferation and migration [42].

In contrast to EphA2, it has been shown that an overexpression of EphB1 was associated with a favourable prognosis for glioma patients. This, however, was dependent on stimulation by its ligand, ephrin-B2, with this interaction resulting in an inhibition of cell invasion and migration [43]. Conversely, EphB1 seems to act as a tumour promoter in medulloblastoma. Consequently, when EphB1 was knocked down in the DAOY human medulloblastoma cell line, migration was inhibited [44]. This was exemplified by a decrease in the expression of β1-integrin and the levels of phosphorylated Src, two molecules involved in cell adhesion and migration. In addition, it was postulated that EphB1 is important for cell growth and survival as these features were reduced when EphB1 was downregulated, possibly due to reduced cyclin E expression, being a master regulator of cell cycle progression [44]. The levels of proliferating cell nuclear antigen (PCNA) and phosphorylated Akt were also decreased, an indication that the cells were failing to progress from G1 to S phase in the cell cycle. Finally, EGF receptor (EGFR) expression was decreased, indicating that an interaction with EGFR might be involved in EphB1’s oncogenic potential [44]. In glioblastoma, overexpression of EphB2 caused phosphorylation and activation of R-Ras, thereby decreasing cell substrate adhesion and mediating enhanced invasiveness. Signalling via R-Ras also caused the inhibition of the MEK/MAPK pathway, leading to reduced cell growth [45]. Higher expression levels of EphB2 have also been measured in medulloblastoma samples, concomitant with decreased cell adhesion and increased invasion when EphB2 was stimulated with ephrin-B1 [46]. This was substantiated by the observation that targeting the EphB2 receptor via knockdown, combined with radiation, decreased the viability and invasion of medulloblastoma cells [74]. Higher expression of EphB4 and its ligand, ephrin-B2, has also been reported in gliomas and this correlated with a worse prognosis for glioblastoma patients [47].

## 8. EphA2 Plays a Dual Role in Kaposi’s Sarcoma Oncogenesis

Kaposi’s sarcoma, a vascular tumour of endothelial origin, is one of the malignancies associated with the oncogenic Kaposi’s sarcoma-associated herpesvirus (KSHV). KSHV infection of endothelial cells is mediated via interactions between the virus and several cell surface receptors [75,76,77]. Some of the receptors implicated in the KSHV entry process include integrins (α3β1, αVβ3, and αVβ5), the xCT cystine/glutamate reporter and EphA2, with heparan sulphate (HS) being a major cell attachment factor. Indeed, KSHV first attaches to HS and then interacts with the integrins and xCT, causing the initiation of a signalling cascade via phosphorylation of focal adhesion kinase (FAK), Src and PI3K, and the recruitment of the adaptor protein c-Cbl. This is followed by translocation of KSHV into lipid rafts where it interacts with EphA2, causing amplification of the signalling cascade. The association of c-Cbl and myosin IIA with EphA2 causes bleb formation and micropinocytosis of KSHV [78,79]. EphA2 is essential for viral entry, as knockdown or deletion of EphA2 abolished the infection of endothelial cells [48,78]. Furthermore, it is EphA2’s intracellular Pkinase-Tyr domain that is important for KSHV infection, shown by the fact that overexpression of full-length EphA2 but not a mutant lacking this domain resulted in enhanced KSHV infection [48]. There is evidence that EphA4 may also be an entry receptor for KSHV; EphA4 expression has been detected in KSHV target cells and an EphA2-EphA4 double-knockout resulted in a greater reduction in infection than EphA2 or EphA4 single knockouts [80]. Another Eph receptor possibly involved in KSHV infection is EphA5 as transduction of an EphA5 (or an EphA4) construct was able to rescue infection in cells that were deficient for EphA2 [81].

From this, it is evident that EphA2 plays a key role in KSHV entry, and this suggests that it may also be an important determinant for susceptibility to KS. Indeed, EphA2 is upregulated in a number of cancers (see Table 1) and, likewise, immunohistochemistry has demonstrated increased protein expression of EphA2 in KS skin tissue [48]. While KSHV infection is necessary and HIV infection and/or other forms of immune suppression are important contributing factors for the development of KSHV-associated cancers, not all KSHV/HIV-co-infected individuals develop KS. Additionally, the rates of KSHV infection do not necessarily correlate with rates of KSHV exposure. This suggests that the processes of KSHV infection and KS progression may also have host genetic components to them. Previous work from our laboratory found that EphA2 sequence variants were associated both with KSHV infection and KS prevalence in HIV-infected patients [82]. These variants were identified in the intracellular and functionally important Pkinase-Tyr and SAM domains. While Pkinase-Tyr variations were associated with KS may be due to enhanced EphA2 Pkinase-Tyr signalling, variations associated with susceptibility to KSHV infection were hypothesised to be related to an enhancement in the EphA2 signalling downstream of KSHV binding necessary for its internalisation. Altered SAM functioning is likely behind the association between variations in the SAM domain and KS because of this region’s role in mediating signalling downstream of EphA2 activation through the binding of adaptor proteins [82].

## 9. EphA2 and Oncogenesis

**Figure 2:**
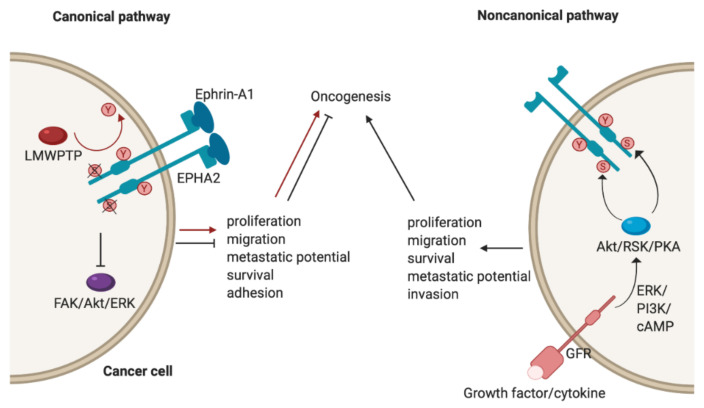
The canonical and noncanonical oncogenic mechanisms of EphA2. Phosphorylated residues are indicated with red circles; Y = tyrosine, S = serine. Figure created with BioRender.com.

As highlighted above, EphA2 plays an important role in a number of cancers (see Table 1); however, its role is context-dependent, and it can act as either a tumour promoter or tumour suppressor. There has been an accumulation of evidence that shows that EphA2 possesses peculiar modes of signalling and it may be that these underscore its opposing functions [4]. As already alluded to (see Section 3), EphA2 has both canonical and noncanonical modes of driving oncogenesis (Figure 2). The canonical pathway involves ligand- and tyrosine kinase-dependent forward signalling via EphA2, which suppresses tumorigenesis. It does so through the inhibition of FAK, Akt and ERK phosphorylation, thereby controlling cell motility and survival [4]. For example, upon EphA2 autophosphorylation, it can no longer associate with FAK to cause phosphorylation and activation. FAK has been implicated in the growth of breast cancer cell lines and its deactivation resulted in reduced oncogenic activity [10]. Importantly, this mechanism is specifically reliant on Tyr772 phosphorylation, highlighted by the fact that a phosphorylation-abrogating Tyr772 mutation resulted in increased transendothelial migration [83]. Consequently, a low level of EphA2 forward signalling promotes tumorigenicity. As already mentioned in the context of breast cancer, this could be due to low ephrin expression, an impaired EphA2-ephrin-A1 interaction due to loss of cadherin, or dephosphorylation of Tyr772 by LMW-PTP [8,51,52]. A good example of this duality has been identified with regards to mesothelioma. Here, EphA2 activation by ephrin-A1 is associated with suppressed tumorigenesis. However, in mesothelioma cell lines, EphA2 was found to be overexpressed, and therefore, in the absence of sufficient ligand, the signalling of other RTKs through the Ras oncogene results in the promotion of malignancy [84].

The noncanonical pathway, conversely, involves the ligand- and tyrosine kinase-independent activation and phosphorylation of EphA2. This is regulated by inflammatory cytokines and growth factors via phosphorylation of EphA2 at Ser897, with induction of this phosphorylation carried out by RSK, Akt and protein kinase A (PKA) [4]. The effects of this phosphorylation include localisation of EphA2 at the leading edge of migrating cells, allowing for actin cytoskeleton assembly and the formation of lamellipodia [71], thereby leading to the promotion and maintenance of certain cancer cell features such as motility and proliferation [4]. A recent demonstration of the noncanonical EphA2 action was focused on its role in the oncogenesis of bladder cancer. Here, the growth factor progranulin was found to be the predominant EphA2 ligand and was upregulated compared to ephrin-A1, which was expressed at normal levels. Stimulation of EphA2 by progranulin resulted in Ser897 phosphorylation, allowing for interaction with liprin-α1, a protein that is necessary for cell motility to occur in this case [85].

## 10. Conclusions

Eph receptors play a complex role in oncogenesis, which is dependent on the specific interactions between the receptors, ligands, signalling pathways and adaptor proteins. Moreover, Eph receptors are implicated in tumour promotion and suppression, depending on the context—their differential expression having consequences for cell proliferation and migration [2]. It is important that the effects of each receptor are investigated in a cancer-specific manner as it is evident that Eph receptors do not always function the same way; EphA7, e.g., has a tumour-suppressive role in colorectal cancer and prostate cancer, but is tumour-promoting in NSCLC [35]. Temporal context is also an important consideration as it is possible for there to be different levels of expression for different stages of disease such as is the case for EphA1 in colorectal and prostate cancer [35]. While the involvement of these receptors in breast, colorectal, lung, prostate and brain cancer has been outlined here, their role in other cancers cannot be excluded and warrants further study. Additionally, further research is required to determine the prognostic value of the Eph receptors and how they can be potentially targeted in novel cancer therapies. EphA2, in particular, is a major receptor of interest, in no small part due to it having both canonical and noncanonical oncogenic mechanisms. While EphA2 has been well characterised in the oncogenesis of breast cancer, its association with lesser prevalent cancers such as KS is still being elucidated. So far, EphA2 sequence variations in KS patients have been identified and their functional significance investigated, but the consequences for KS oncogenesis are not yet fully understood [82]. Therefore, subsequent research into EphA2’s role in KS development is required, which will potentially also shed light on the oncogenic role of EphA2 in other cancers. In conclusion, Eph receptors present an important and promising area of study in the oncogenesis of a variety of cancers and may have important clinical implications.

## Figures and Tables

**Table 1:** Summary of the various Eph receptors and their implicated roles depending on cancer type.

Cancer Type	Eph Receptor	Aberrant Function	Role of Eph Receptor	Reference
Breast cancer	EphA2	Overexpressed; impaired tyrosine phosphorylation	Tumour-promoting	[8,10]
	EphA4	Overexpressed	Tumour-promoting	[11]
	EphA5	Low expression	Tumour-suppressive	[12]
	EphA7	Overexpressed	Tumour-promoting	[11]
	EphA10	Overexpressed	Tumour-promoting	[13]
	EphB2	Overexpressed	Tumour-promoting	[14]
	EphB4	Overexpressed/low expression	Tumour-promoting/ tumour-suppressive	[2]
	EphB6	Low expression	Tumour-suppressive	[15]
Colorectal cancer	EphA1	Overexpressed in early stages; low expression in later stages	Tumour-suppressive	[16]
	EphA2	Overexpressed in early stages; low expression in later stages	Tumour-suppressive	[16]
	EphA3	Low expression	Tumour-suppressive	[17]
	EphA4	Overexpressed	Tumour-promoting	[18]
	EphA7	Low expression	Tumour-suppressive	[19]
	EphB2	Overexpressed in early stages; low expression in later stages	Tumour-suppressive	[20,21]
	EphB3	Overexpressed in early stages; low expression in later stages	Tumour-suppressive	[20,21]
	EphB4	Overexpressed	Tumour-promoting	[19]
	EphB6	Low expression	Tumour-suppressive	[22,23]
Lung cancer	EphA1	Overexpressed	Tumour-promoting	[24]
	EphA2	Increased expression in advanced stages	Tumour-promoting	[25]
	EphA3	Low expression	Tumour-suppressive	[26]
	EphA4	Overexpressed	Tumour-promoting	[24]
	EphA5	Overexpressed	Tumour-promoting	[24]
	EphA7	Overexpressed	Tumour-promoting	[24]
	EphB3	Overexpressed	Tumour-promoting	[27]
	EphB4	Overexpressed	Tumour-promoting	[28]
	EphB6	Low expression	Tumour-suppressive	[29,30]
Prostate cancer	EphA1	Low expression	Tumour-suppressive	[6,31]
	EphA2	Overexpressed	Tumour-promoting	[32]
	EphA3	Overexpressed	Tumour-promoting	[31]
	EphA4	Overexpressed	Tumour-promoting	[33]
	EphA5	Low expression	Tumour-suppressive	[34]
	EphA6	Overexpressed	Tumour-promoting	[35,36]
	EphA7	Low expression	Tumour-suppressive	[37]
	EphB2	Mutational inactivation	Tumour-suppressive	[38]
	EphB3	Overexpressed	Tumour-promoting	[39]
	EphB4	Overexpressed	Tumour-promoting	[39]
Brain cancer	EphA2	Overexpressed in glioblastoma	Tumour-promoting	[40]
	EphA3	Overexpressed in glioblastoma	Tumour-promoting	[41]
	EphA4	Overexpressed in glioblastoma	Tumour-promoting	[42]
	EphB1	Low expression in glioblastoma	Tumour-suppressive; dependent on ligand stimulation	[43]
		Overexpressed in medulloblastoma	Tumour-promoting	[44]
	EphB2	Overexpressed in glioblastoma	Tumour-promoting	[45]
		Overexpressed in medulloblastoma		[46]
	EphB4	Overexpressed in glioblastoma	Tumour-promoting	[47]
Kaposi’s sarcoma	EphA2	Overexpressed; impaired tyrosine phosphorylation	Unknown	[48]

## Data Availability

Data sharing not applicable.

## References

[1] Genander M., Frisen J. (2010). Ephrins and Eph receptors in stem cells and cancer. Curr. Opin. Cell Biol..

[2] Kou C.J., Kandpal R.P. (2018). Differential Expression Patterns of Eph Receptors and Ephrin Ligands in Human Cancers. BioMed Res. Int..

[3] Mesri E.A., Cesarman E., Boshoff C. (2010). Kaposi’s sarcoma and its associated herpesvirus. Nat. Rev. Cancer.

[4] Zhou Y., Sakurai H. (2017). Emerging and Diverse Functions of the EphA2 Noncanonical Pathway in Cancer Progression. Biol. Pharm. Bull..

[5] Surawska H., Ma P.C., Salgia R. (2004). The role of ephrins and Eph receptors in cancer. Cytokine Growth Factor Rev..

[6] Castano J., Davalos V., Schwartz S., Arango D. (2008). EPH receptors in cancer. Histol. Histopathol..

[7] Pasquale E.B. (2005). Eph receptor signalling casts a wide net on cell behaviour. Nat. Rev. Mol. Cell Biol..

[8] Pasquale E.B. (2010). Eph receptors and ephrins in cancer: Bidirectional signalling and beyond. Nat. Rev. Cancer.

[9] Pasquale E.B. (2008). Eph-ephrin bidirectional signaling in physiology and disease. Cell.

[10] Nakamoto M., Bergemann A.D. (2002). Diverse roles for the Eph family of receptor tyrosine kinases in carcinogenesis. Microsc. Res. Tech..

[11] Brantley-Sieders D.M., Jiang A., Sarma K., Badu-Nkansah A., Walter D.L., Shyr Y., Chen J. (2011). Eph/ephrin profiling in human breast cancer reveals significant associations between expression level and clinical outcome. PLoS ONE.

[12] Fu D.Y., Wang Z.M., Wang B.L., Chen L., Yang W.T., Shen Z.Z., Huang W., Shao Z.M. (2010). Frequent epigenetic inactivation of the receptor tyrosine kinase EphA5 by promoter methylation in human breast cancer. Hum. Pathol..

[13] Nagano K., Kanasaki S., Yamashita T., Maeda Y., Inoue M., Higashisaka K., Yoshioka Y., Abe Y., Mukai Y., Kamada H. (2013). Expression of Eph receptor A10 is correlated with lymph node metastasis and stage progression in breast cancer patients. Cancer Med..

[14] Husa A.M., Magic Z., Larsson M., Fornander T., Perez-Tenorio G. (2016). EPH/ephrin profile and EPHB2 expression predicts patient survival in breast cancer. Oncotarget.

[15] Fox B.P., Kandpal R.P. (2006). Transcriptional silencing of EphB6 receptor tyrosine kinase in invasive breast carcinoma cells and detection of methylated promoter by methylation specific PCR. Biochem. Biophys. Res. Commun..

[16] Herath N.I., Doecke J., Spanevello M.D., Leggett B.A., Boyd A.W. (2009). Epigenetic silencing of EphA1 expression in colorectal cancer is correlated with poor survival. Br. J. Cancer.

[17] Wang Y., Xuan Z., Wang B., Zhang D., Zhang C., Wang J., Sun Y. (2019). EphA3 Downregulation by Hypermethylation Associated with Lymph Node Metastasis and TNM Stage in Colorectal Cancer. Dig. Dis. Sci..

[18] De Marcondes P.G., Bastos L.G., de-Freitas-Junior J.C., Rocha M.R., Morgado-Diaz J.A. (2016). EphA4-mediated signaling regulates the aggressive phenotype of irradiation survivor colorectal cancer cells. Tumour Biol..

[19] Herath N.I., Spanevello M.D., Doecke J.D., Smith F.M., Pouponnot C., Boyd A.W. (2012). Complex expression patterns of Eph receptor tyrosine kinases and their ephrin ligands in colorectal carcinogenesis. Eur. J. Cancer.

[20] Batlle E., Bacani J., Begthel H., Jonkheer S., Gregorieff A., van de Born M., Malats N., Sancho E., Boon E., Pawson T. (2005). EphB receptor activity suppresses colorectal cancer progression. Nature.

[21] Cortina C., Palomo-Ponce S., Iglesias M., Fernandez-Masip J.L., Vivancos A., Whissell G., Huma M., Peiro N., Gallego L., Jonkheer S. (2007). EphB-ephrin-B interactions suppress colorectal cancer progression by compartmentalizing tumor cells. Nat. Genet..

[22] Mateo-Lozano S., Bazzocco S., Rodrigues P., Mazzolini R., Andretta E., Dopeso H., Fernandez Y., Del Llano E., Bilic J., Suarez-Lopez L. (2017). Loss of the EPH receptor B6 contributes to colorectal cancer metastasis. Sci. Rep..

[23] Peng L., Tu P., Wang X., Shi S., Zhou X., Wang J. (2014). Loss of EphB6 protein expression in human colorectal cancer correlates with poor prognosis. J. Mol. Histol..

[24] Giaginis C., Tsoukalas N., Bournakis E., Alexandrou P., Kavantzas N., Patsouris E., Theocharis S. (2014). Ephrin (Eph) receptor A1, A4, A5 and A7 expression in human non-small cell lung carcinoma: Associations with clinicopathological parameters, tumor proliferative capacity and patients’ survival. BMC Clin. Pathol..

[25] Kinch M.S., Moore M.B., Harpole D.H. (2003). Predictive value of the EphA2 receptor tyrosine kinase in lung cancer recurrence and survival. Clin. Cancer Res..

[26] Peng J., Wang Q., Liu H., Ye M., Wu X., Guo L. (2016). EPHA3 regulates the multidrug resistance of small cell lung cancer via the PI3K/BMX/STAT3 signaling pathway. Tumour Biol..

[27] Ji X.D., Li G., Feng Y.X., Zhao J.S., Li J.J., Sun Z.J., Shi S., Deng Y.Z., Xu J.F., Zhu Y.Q. (2011). EphB3 is overexpressed in non-small-cell lung cancer and promotes tumor metastasis by enhancing cell survival and migration. Cancer Res..

[28] Ferguson B.D., Liu R., Rolle C.E., Tan Y.H., Krasnoperov V., Kanteti R., Tretiakova M.S., Cervantes G.M., Hasina R., Hseu R.D. (2013). The EphB4 receptor tyrosine kinase promotes lung cancer growth: A potential novel therapeutic target. PLoS ONE.

[29] Bulk E., Yu J., Hascher A., Koschmieder S., Wiewrodt R., Krug U., Timmermann B., Marra A., Hillejan L., Wiebe K. (2012). Mutations of the EPHB6 receptor tyrosine kinase induce a pro-metastatic phenotype in non-small cell lung cancer. PLoS ONE.

[30] Yu J., Bulk E., Ji P., Hascher A., Tang M., Metzger R., Marra A., Serve H., Berdel W.E., Wiewroth R. (2010). The EPHB6 receptor tyrosine kinase is a metastasis suppressor that is frequently silenced by promoter DNA hypermethylation in non-small cell lung cancer. Clin. Cancer Res..

[31] Fox B.P., Tabone C.J., Kandpal R.P. (2006). Potential clinical relevance of Eph receptors and ephrin ligands expressed in prostate carcinoma cell lines. Biochem. Biophys. Res. Commun..

[32] Zeng G., Hu Z., Kinch M.S., Pan C.X., Flockhart D.A., Kao C., Gardner T.A., Zhang S., Li L., Baldridge L.A. (2003). High-level expression of EphA2 receptor tyrosine kinase in prostatic intraepithelial neoplasia. Am. J. Pathol..

[33] Ashida S., Nakagawa H., Katagiri T., Furihata M., Iiizumi M., Anazawa Y., Tsunoda T., Takata R., Kasahara K., Miki T. (2004). Molecular features of the transition from prostatic intraepithelial neoplasia (PIN) to prostate cancer: Genome-wide gene-expression profiles of prostate cancers and PINs. Cancer Res..

[34] Li S., Zhu Y., Ma C., Qiu Z., Zhang X., Kang Z., Wu Z., Wang H., Xu X., Zhang H. (2015). Downregulation of EphA5 by promoter methylation in human prostate cancer. BMC Cancer.

[35] Buckens O.J., El Hassouni B., Giovannetti E., Peters G.J. (2020). The role of Eph receptors in cancer and how to target them: Novel approaches in cancer treatment. Expert Opin. Investig. Drugs.

[36] Li S., Ma Y., Xie C., Wu Z., Kang Z., Fang Z., Su B., Guan M. (2015). EphA6 promotes angiogenesis and prostate cancer metastasis and is associated with human prostate cancer progression. Oncotarget.

[37] Guan M., Xu C., Zhang F., Ye C. (2009). Aberrant methylation of EphA7 in human prostate cancer and its relation to clinicopathologic features. Int. J. Cancer.

[38] Huusko P., Ponciano-Jackson D., Wolf M., Kiefer J.A., Azorsa D.O., Tuzmen S., Weaver D., Robbins C., Moses T., Allinen M. (2004). Nonsense-mediated decay microarray analysis identifies mutations of EPHB2 in human prostate cancer. Nat. Genet..

[39] Astin J.W., Batson J., Kadir S., Charlet J., Persad R.A., Gillatt D., Oxley J.D., Nobes C.D. (2010). Competition amongst Eph receptors regulates contact inhibition of locomotion and invasiveness in prostate cancer cells. Nat. Cell Biol..

[40] Hamaoka Y., Negishi M., Katoh H. (2016). EphA2 is a key effector of the MEK/ERK/RSK pathway regulating glioblastoma cell proliferation. Cell. Signal..

[41] Day B.W., Stringer B.W., Al-Ejeh F., Ting M.J., Wilson J., Ensbey K.S., Jamieson P.R., Bruce Z.C., Lim Y.C., Offenhauser C. (2013). EphA3 maintains tumorigenicity and is a therapeutic target in glioblastoma multiforme. Cancer Cell.

[42] Fukai J., Yokote H., Yamanaka R., Arao T., Nishio K., Itakura T. (2008). EphA4 promotes cell proliferation and migration through a novel EphA4-FGFR1 signaling pathway in the human glioma U251 cell line. Mol. Cancer Ther..

[43] Teng L., Nakada M., Furuyama N., Sabit H., Furuta T., Hayashi Y., Takino T., Dong Y., Sato H., Sai Y. (2013). Ligand-dependent EphB1 signaling suppresses glioma invasion and correlates with patient survival. Neuro Oncol..

[44] Bhatia S., Baig N.A., Timofeeva O., Pasquale E.B., Hirsch K., MacDonald T.J., Dritschilo A., Lee Y.C., Henkemeyer M., Rood B. (2015). Knockdown of EphB1 receptor decreases medulloblastoma cell growth and migration and increases cellular radiosensitization. Oncotarget.

[45] Nakada M., Niska J.A., Tran N.L., McDonough W.S., Berens M.E. (2005). EphB2/R-Ras signaling regulates glioma cell adhesion, growth, and invasion. Am. J. Pathol..

[46] Sikkema A.H., den Dunnen W.F., Hulleman E., van Vuurden D.G., Garcia-Manero G., Yang H., Scherpen F.J., Kampen K.R., Hoving E.W., Kamps W.A. (2012). EphB2 activity plays a pivotal role in pediatric medulloblastoma cell adhesion and invasion. Neuro Oncol..

[47] Tu Y., He S., Fu J., Li G., Xu R., Lu H., Deng J. (2012). Expression of EphrinB2 and EphB4 in glioma tissues correlated to the progression of glioma and the prognosis of glioblastoma patients. Clin. Transl. Oncol..

[48] Hahn A.S., Kaufmann J.K., Wies E., Naschberger E., Panteleev-Ivlev J., Schmidt K., Holzer A., Schmidt M., Chen J., Konig S. (2012). The ephrin receptor tyrosine kinase A2 is a cellular receptor for Kaposi’s sarcoma-associated herpesvirus. Nat. Med..

[49] Brantley-Sieders D.M., Zhuang G., Hicks D., Fang W.B., Hwang Y., Cates J.M., Coffman K., Jackson D., Bruckheimer E., Muraoka-Cook R.S. (2008). The receptor tyrosine kinase EphA2 promotes mammary adenocarcinoma tumorigenesis and metastatic progression in mice by amplifying ErbB2 signaling. J. Clin. Investig..

[50] Zelinski D.P., Zantek N.D., Stewart J.C., Irizarry A.R., Kinch M.S. (2001). EphA2 overexpression causes tumorigenesis of mammary epithelial cells. Cancer Res..

[51] Kikawa K.D., Vidale D.R., Van Etten R.L., Kinch M.S. (2002). Regulation of the EphA2 kinase by the low molecular weight tyrosine phosphatase induces transformation. J. Biol. Chem..

[52] Zantek N.D., Azimi M., Fedor-Chaiken M., Wang B., Brackenbury R., Kinch M.S. (1999). E-cadherin regulates the function of the EphA2 receptor tyrosine kinase. Cell Growth Differ..

[53] Dong Y., Liu Y., Jiang A., Li R., Yin M., Wang Y. (2018). MicroRNA-335 suppresses the proliferation, migration, and invasion of breast cancer cells by targeting EphA4. Mol. Cell. Biochem..

[54] Hachim I.Y., Villatoro M., Canaff L., Hachim M.Y., Boudreault J., Haiub H., Ali S., Lebrun J.J. (2017). Transforming Growth Factor-beta Regulation of Ephrin Type-A Receptor 4 Signaling in Breast Cancer Cellular Migration. Sci. Rep..

[55] Johnson C., Segovia B., Kandpal R.P. (2016). EPHA7 and EPHA10 Physically Interact and Differentially Co-localize in Normal Breast and Breast Carcinoma Cell Lines, and the Co-localization Pattern Is Altered in EPHB6-expressing MDA-MB-231 Cells. Cancer Genom. Proteom..

[56] Noren N.K., Foos G., Hauser C.A., Pasquale E.B. (2006). The EphB4 receptor suppresses breast cancer cell tumorigenicity through an Abl-Crk pathway. Nat. Cell Biol..

[57] Merlos-Suarez A., Batlle E. (2008). Eph-ephrin signalling in adult tissues and cancer. Curr. Opin. Cell Biol..

[58] Rutkowski R., Mertens-Walker I., Lisle J.E., Herington A.C., Stephenson S.A. (2012). Evidence for a dual function of EphB4 as tumor promoter and suppressor regulated by the absence or presence of the ephrin-B2 ligand. Int. J. Cancer.

[59] Noren N.K., Yang N.Y., Silldorff M., Mutyala R., Pasquale E.B. (2009). Ephrin-independent regulation of cell substrate adhesion by the EphB4 receptor. Biochem. J..

[60] Xiao Z., Carrasco R., Kinneer K., Sabol D., Jallal B., Coats S., Tice D.A. (2012). EphB4 promotes or suppresses Ras/MEK/ERK pathway in a context-dependent manner: Implications for EphB4 as a cancer target. Cancer Biol. Ther..

[61] Lam S., Wiercinska E., Teunisse A.F., Lodder K., ten Dijke P., Jochemsen A.G. (2014). Wild-type p53 inhibits pro-invasive properties of TGF-beta3 in breast cancer, in part through regulation of EPHB2, a new TGF-beta target gene. Breast Cancer Res. Treat..

[62] Chukkapalli S., Amessou M., Dilly A.K., Dekhil H., Zhao J., Liu Q., Bejna A., Thomas R.D., Bandyopadhyay S., Bismar T.A. (2014). Role of the EphB2 receptor in autophagy, apoptosis and invasion in human breast cancer cells. Exp. Cell Res..

[63] Wu B.O., Jiang W.G., Zhou D., Cui Y.X. (2016). Knockdown of EPHA1 by CRISPR/CAS9 Promotes Adhesion and Motility of HRT18 Colorectal Carcinoma Cells. Anticancer Res..

[64] Kadife E., Ware T.M.B., Luwor R.B., Chan S.T.F., Nurgali K., Senior P.V. (2018). Effects of EphB4 receptor expression on colorectal cancer cells, tumor growth, vascularization and composition. Acta Oncol..

[65] Faoro L., Singleton P.A., Cervantes G.M., Lennon F.E., Choong N.W., Kanteti R., Ferguson B.D., Husain A.N., Tretiakova M.S., Ramnath N. (2010). EphA2 mutation in lung squamous cell carcinoma promotes increased cell survival, cell invasion, focal adhesions, and mammalian target of rapamycin activation. J. Biol. Chem..

[66] Li G., Ji X.D., Gao H., Zhao J.S., Xu J.F., Sun Z.J., Deng Y.Z., Shi S., Feng Y.X., Zhu Y.Q. (2012). EphB3 suppresses non-small-cell lung cancer metastasis via a PP2A/RACK1/Akt signalling complex. Nat. Commun..

[67] Muller-Tidow C., Diederichs S., Bulk E., Pohle T., Steffen B., Schwable J., Plewka S., Thomas M., Metzger R., Schneider P.M. (2005). Identification of metastasis-associated receptor tyrosine kinases in non-small cell lung cancer. Cancer Res..

[68] Soler M., Mancini F., Meca-Cortes O., Sanchez-Cid L., Rubio N., Lopez-Fernandez S., Lozano J.J., Blanco J., Fernandez P.L., Thomson T.M. (2009). HER3 is required for the maintenance of neuregulin-dependent and -independent attributes of malignant progression in prostate cancer cells. Int. J. Cancer.

[69] Li S., Wu Z., Ma P., Xu Y., Chen Y., Wang H., He P., Kang Z., Yin L., Zhao Y. (2017). Ligand-dependent EphA7 signaling inhibits prostate tumor growth and progression. Cell Death Dis..

[70] Mertens-Walker I., Fernandini B.C., Maharaj M.S., Rockstroh A., Nelson C.C., Herington A.C., Stephenson S.A. (2015). The tumour-promoting receptor tyrosine kinase, EphB4, regulates expression of integrin-beta8 in prostate cancer cells. BMC Cancer.

[71] Miao H., Li D.Q., Mukherjee A., Guo H., Petty A., Cutter J., Basilion J.P., Sedor J., Wu J., Danielpour D. (2009). EphA2 mediates ligand-dependent inhibition and ligand-independent promotion of cell migration and invasion via a reciprocal regulatory loop with Akt. Cancer Cell.

[72] Binda E., Visioli A., Giani F., Lamorte G., Copetti M., Pitter K.L., Huse J.T., Cajola L., Zanetti N., DiMeco F. (2012). The EphA2 receptor drives self-renewal and tumorigenicity in stem-like tumor-propagating cells from human glioblastomas. Cancer Cell.

[73] Wang S.Y., Yu L., Ling G.Q., Xiao S., Sun X.L., Song Z.H., Liu Y.J., Jiang X.D., Cai Y.Q., Ke Y.Q. (2012). Vasculogenic mimicry and its clinical significance in medulloblastoma. Cancer Biol. Ther..

[74] Bhatia S., Hirsch K., Bukkapatnam S., Baig N.A., Oweida A., Griego A., Calame D., Sharma J., Donson A., Foreman N. (2017). Combined EphB2 receptor knockdown with radiation decreases cell viability and invasion in medulloblastoma. Cancer Cell Int..

[75] Boshoff C. (2012). Ephrin receptor: A door to KSHV infection. Nat. Med..

[76] Chandran B. (2010). Early events in Kaposi’s sarcoma-associated herpesvirus infection of target cells. J. Virol..

[77] Veettil M.V., Bandyopadhyay C., Dutta D., Chandran B. (2014). Interaction of KSHV with host cell surface receptors and cell entry. Viruses.

[78] Chakraborty S., Veettil M.V., Bottero V., Chandran B. (2012). Kaposi’s sarcoma-associated herpesvirus interacts with EphrinA2 receptor to amplify signaling essential for productive infection. Proc. Natl. Acad. Sci. USA.

[79] Kumar B., Chandran B. (2016). KSHV Entry and Trafficking in Target Cells-Hijacking of Cell Signal Pathways, Actin and Membrane Dynamics. Viruses.

[80] Chen J., Zhang X., Schaller S., Jardetzky T.S., Longnecker R. (2019). Ephrin Receptor A4 is a New Kaposi’s Sarcoma-Associated Herpesvirus Virus Entry Receptor. MBio.

[81] TerBush A.A., Hafkamp F., Lee H.J., Coscoy L. (2018). A Kaposi’s Sarcoma-Associated Herpesvirus Infection Mechanism Is Independent of Integrins alpha3beta1, alphaVbeta3, and alphaVbeta5. J. Virol..

[82] Blumenthal M.J., Schutz C., Meintjes G., Mohamed Z., Mendelson M., Ambler J.M., Whitby D., Mackelprang R.D., Carse S., Katz A.A. (2018). EPHA2 sequence variants are associated with susceptibility to Kaposi’s sarcoma-associated herpesvirus infection and Kaposi’s sarcoma prevalence in HIV-infected patients. Cancer Epidemiol..

[83] Locard-Paulet M., Lim L., Veluscek G., McMahon K., Sinclair J., van Weverwijk A., Worboys J.D., Yuan Y., Isacke C.M., Jorgensen C. (2016). Phosphoproteomic analysis of interacting tumor and endothelial cells identifies regulatory mechanisms of transendothelial migration. Sci. Signal..

[84] Nasreen N., Khodayari N., Mohammed K.A. (2012). Advances in malignant pleural mesothelioma therapy: Targeting EphA2 a novel approach. Am. J. Cancer Res..

[85] Buraschi S., Neill T., Xu S.Q., Palladino C., Belfiore A., Iozzo R.V., Morrione A. (2020). Progranulin/EphA2 axis: A novel oncogenic mechanism in bladder cancer. Matrix Biol..

